# Vaginal and vulvar cancer patient experiences of the information pathway from pre-diagnosis to treatment

**DOI:** 10.1007/s00520-026-11022-0

**Published:** 2026-07-21

**Authors:** Tracey DiSipio, Britta Wigginton, Joan Cunningham, Susan Jordan, Abbey Diaz

**Affiliations:** 1 School of Public Health, The University of Queensland, 288 Herston Road, Brisbane, QLD 4006 Australia; 2 Menzies School of Health Research, Charles Darwin University, Darwin, NT Australia; 3 Yardhura Walani National Centre for Aboriginal and Torres Strait Islander Wellbeing Research, National Centre for Epidemiology and Population Health, Australian National University, Canberra, Australia

**Keywords:** Information needs, Information satisfaction, Rare cancer, Supportive care, Vaginal cancer, Vulvar cancer

## Abstract

**Purpose:**

To describe the information experiences of vaginal and vulvar cancer patients, including satisfaction, needs, and preferred sources at three key touchpoints: pre-diagnosis, diagnosis, and treatment.

**Methods:**

This cross-sectional mixed methods study recruited women aged 18+ years living in Queensland, Australia, and diagnosed with primary vaginal or vulvar cancer between 01 June 2022 and 31 August 2023. We obtained self-reports of satisfaction with cancer information (Satisfaction with Cancer Information Profile, SCIP-B, range 7–35), need for information (study-specific measure designed in consultation with consumers including six items from the health system and information domain of the Supportive Care Needs Survey-Short Form, SCNS-SF34), and preferred sources of receiving information (Health Information National Trends Survey, HINTS). Participant-informed recommendations to improve information provision were derived from qualitative interviews.

**Results:**

Of the 39 women who completed the quantitative questionnaire, 16 also participated in a qualitative interview. Mean scores for satisfaction with information increased from pre-diagnosis (20.8, SD 7.1) to during diagnosis (24.0, SD 8.4) and remained stable during treatment (24.8, SD 8.2). Approximately three-quarters of participants reported at least one moderate-to-high unmet information need at each touchpoint. Doctors, internet searches, and family/friends were the preferred information sources. Seventeen recommendations were developed relevant to pre-diagnosis (*n* = 2), diagnosis (*n* = 2), treatment (*n* = 4), post-treatment (*n* = 2), and across touchpoints (*n* = 7).

**Conclusions:**

Dissatisfaction with information and unmet information needs were prevalent among our participants diagnosed with vaginal or vulvar cancer. A variety of participant-informed recommendations were developed which can guide the improvement of information experiences across the cancer care continuum.

**Supplementary Information:**

The online version contains supplementary material available at 10.1007/s00520-026-11022-0.

## Introduction

The Australian Cancer Plan prioritises person-centred care across the cancer care continuum, from early detection through to survivorship [1]. Enhancing the consumer experience by improving information access, and facilitating clear communication tailored to individual needs, is a key objective of the Plan.

Vaginal and vulvar cancers are rare gynaecological cancers, with approximately 500 Australian women diagnosed in 2025 [2]. There is little Australian evidence on women’s experience of these cancers across the cancer care pathway. However, our qualitative study showed that women with vulvar cancer can have difficult diagnostic experiences and lack support and information from diagnosis onward [3].

While Australian research indicates that cancer patients are generally provided with good generic information [4], this may be inadequate to meet the needs of patients with rare cancers [5]. These patients more frequently report unmet information needs throughout their disease journey than those with common cancers, so information tailored to factors including the cancer type and disease phase has been recommended [5]. Previous studies have shown that when satisfaction with information is high and needs are met, patients feel more able to make informed treatment decisions and have enhanced psychosocial outcomes, such as reduced depression, anxiety, and uncertainty and increased quality of life and sense of personal control [6–10]. To ensure patients have the information they require, we need to understand current information provision experiences.

The Model of Pathways to Treatment [11] is a conceptual framework that describes cancer patients’ interactions with the healthcare system around the events (e.g. detection of bodily changes; first consultation with a healthcare professional) and processes (e.g. patient appraisal and self-management; healthcare professional appraisal, investigations, and referrals) from pre-diagnosis through to cancer treatment. These interactions are referred to as touchpoints. The aim of this study was to describe vaginal and vulvar cancer patient information experiences including satisfaction, needs, and sources at three key touchpoints: (1) pre-diagnosis, before first seeing a doctor (including the symptoms experienced, if any); (2) diagnosis, during the diagnostic process (including visiting doctor(s), specialist(s), undergoing examinations and tests, and receiving a cancer diagnosis); and (3) treatment, during the time of planning and receiving cancer treatment (e.g. surgery, radiotherapy, chemotherapy). Drawing on participant interviews, we also developed recommendations to improve information provision at each touchpoint.

## Methods

### Study design

This cross-sectional mixed methods study included a mailed self-administered questionnaire and subsequent semi-structured telephone interviews among women diagnosed with vaginal and vulvar cancers in Queensland, Australia. Ethics approval was obtained prior to study commencement from The University of Queensland Human Research Ethics Committee (HE000046). The reporting of our study conforms with the Mixed Methods Reporting in Rehabilitation and Health Sciences (MMR-RHS) checklist [12].

### Participants

It is an Australian legal requirement that all cancer diagnoses (except non-melanoma skin cancers) are notified to state-based cancer registries; hence, we asked Cancer Alliance Queensland (CAQ), which houses the Queensland Cancer Register, to identify all primary vaginal (ICD-10 C52) and vulvar (ICD-10 C51) cancer notifications received between 01/06/2022 and 31/08/2023. After excluding cases aged 81+ years at diagnosis, living in a nursing home, or deceased, CAQ staff mailed eligible potential participants an invitation letter, consent form, and questionnaire with a reply-paid envelope. Non-responders were sent up to two reminders. We also invited participants to be interviewed to provide more details about their experiences. We obtained written consent to participate in the study and to access data from the Queensland Cancer Register.

### Data collection

Participants were asked to complete the questionnaire thinking back to the time before their diagnosis, during diagnosis, and during treatment. Study-specific measures were designed in consultation with consumers and drawing on existing tools and relevant research. Consumers (*n* = 3, including one consumer with a lived experience of vaginal cancer and one with vulvar cancer) tested the questionnaire and provided feedback, and changes were made to non-validated questions to improve the comprehensibility (i.e. clarity and order of questions), relevance (i.e. survey items and response options), acceptability (i.e. ability and willingness to answer questions), and feasibility (i.e. the amount of time taken to complete the survey) of the questionnaire.

We used the Satisfaction with Cancer Information Profile part B (SCIP-B) measure to collect information on participants’ satisfaction with the type and timing of information across seven items on a 5-point Likert scale (from 1 = very dissatisfied to 5 = very satisfied) [13]. Based on consumer feedback, we added two overall measures of satisfaction and helpfulness of information.

We used a study-specific measure to collect data on need for information. Need items were specific to each touchpoint: pre-diagnosis (11 items), during diagnosis (10 items), and during treatment (12 items). Need items included six items from the health system and information domain of the Supportive Care Needs Survey-Short Form (SCNS-SF34): two items during diagnosis, three items during treatment, and one item repeated at both of these touchpoints [14]. Participants rated their level of need for each item on a 5-point Likert scale based on the SCNS-SF34 (from 1 = not applicable to 5 = high unmet need) [14]. For each touchpoint, we asked participants to select the top three preferred sources of information, as measured by the Health Information National Trends Survey (HINTS) [15].

The questionnaire also included sociodemographic (e.g. age) items and clinical factors (e.g. cancer history, cancer treatment). Additionally, we measured health literacy using a 5-point Likert scale (1 = never, 5 = always) using the Single Item Literacy Screener (SILS) which asks, “How often do you need to have someone help you when you read instructions, pamphlets, or other written material from your doctor or pharmacist?” [16]. The scale developers suggest that responses of greater than two on SILS indicate some difficulty with reading printed health-related material [16].

Semi-structured telephone interviews were conducted by BW, a female qualitative researcher trained in psychology who had no prior interaction with participants nor involvement in their care. Interviews were scheduled at a time convenient to participants. Only BW and the participant were present for the interview. Interviews were audio-recorded and transcribed verbatim by a professional transcription service. A topic guide and journey map were used to guide conversation on information experiences, support notetaking, and help ensure all touchpoints were addressed (Supplementary Material 1). For this paper, the focus was on women’s suggestions for changes to information and communication to improve their cancer care experiences. Through analysis and synthesis of these data, investigators derived participant-informed recommendations for improvements to information provision.

For all eligible women, CAQ provided information on primary site of cancer (International Classification of Diseases for Oncology, ICD-0–3.2) [17], disease stage at diagnosis (registry derived stage classified according to the International Federation of Gynaecology and Obstetrics, FIGO) [18, 19], remoteness of residence (classified according to the Australian Standard Geographical Classification, ASGC) [20], and socio-economic status (based on the Socio-Economic Indexes for Areas, SEIFA) [21].

### Data analysis

We summarised participant characteristics using descriptive statistics across women who completed the survey, completed survey and interview, and did not participate, and examined differences between participants and non-participants using chi-squared tests and analysis of variance (ANOVA). In accordance with CAQ requirements, when fewer than five women (*n* < 5) were available for reporting on characteristics, we present an approximate proportion.

As per the SCIP-B manual, where at least four of the seven SCIP-B items were completed, we imputed mean values for the missing items based on that person’s responses to other completed items [13]. If three or fewer SCIP-B items were completed, we did not assign the participant a total SCIP-B score. Total satisfaction scores were calculated by summing the responses, including imputed responses. A possible score ranges from 7 (very dissatisfied) to 35 (very satisfied) [13]. Means and standard deviations (SD) for individual SCIP-B items and total satisfaction are presented at each touchpoint. We also dichotomised responses to individual SCIP-B items as satisfied (4–5) and not satisfied (1–3) with prevalence reported at each touchpoint.

To assess the potential impact of missing data, differences in characteristics of participants with and without a SCIP-B score pre-diagnosis (as the touchpoint with the greatest amount of missing data) were compared. Sensitivity analyses were conducted to compare total SCIP-B scores at each touchpoint (means and SDs) with (a) all available data, (b) complete data, (c) using linear mixed models unadjusted, and (d) adjusted for age. Differences in the four approaches yielded a mean difference of 0.1 to 0.6 units (Supplementary Table 1); therefore, descriptive summary statistics using all available data are reported.

As there is no minimally important difference (MID) for the SCIP-B, we defined this a priori as three points for mean total satisfaction. We based our definition on the MID of the European Organisation for Research and Treatment of Cancer (EORTC) for a 10-point difference (scale 0 to 100), pro-rated to the SCIP-B scale [22].

Each “need for information” item was dichotomised into no-to-low (1 to 3) and moderate-to-high (4 to 5) unmet needs. The prevalence of moderate-to-high unmet need for information at each touchpoint is presented. Responses for each of the preferred sources of receiving information were summed to identify the most prevalent preferences at each touchpoint. All quantitative data analyses were performed with Stata version 19 [23].

Data related to suggestions on what and/or how to improve information provision were extracted. Data provided in response to the interview question, “Were there aspects about the information/communication that you would change if you could?” and relevant data throughout the interviews, were extracted. One investigator (TD) deductively manually coded relevant data. Initial codes relating to information satisfaction, needs, and sources by touchpoint were discussed with BW to develop codes further. Another investigator (AD) independently manually coded interview data using updated codes to ensure completeness. TD and AD discussed coded participant-informed recommendations until consensus was obtained. These data were analysed descriptively using content analysis [24] and synthesised as participant-informed recommendations.

## Results

### Sample

Of the 112 eligible women contacted, 39 women returned a completed questionnaire (35% response). Of those, 16 women expressed interest and subsequently participated in an interview between December 2023 and April 2024. Interviews lasted between 38 and 80 min (median 50 min).

Demographic and disease characteristics were similar for participating (*n* = 39) and non-participating women (*n* = 73) (Supplementary Table 2). Small numbers (*n* < 5) precluded separate reporting for women who identified as First Nations. Characteristics of women with (*n* = 29) and without (*n* = 10) a total SCIP-B score pre-diagnosis were similar (data not shown due to small cell sizes).

Study sample characteristics are presented in Table 1. At the time of survey completion, the median age of participants was 66 years (range 41–82) and they were 11 months (median, range 3–19) post-diagnosis. Over half were married/living with a partner, had completed a high school education or below, and had private health insurance. The majority were born in Australia, had an adequate level of health literacy, no prior cancer diagnoses, reported one or more comorbidities, and were diagnosed with stage I/II disease. The characteristics of women who participated in an interview (*n* = 16) were generally similar to those who completed a survey only (*n* = 23) (data not shown due to small cell sizes).

**Table 1:** Characteristics of study participants

Characteristics	Participants	
	Median	Minimum, maximum
Age in years at survey	66	(41, 82)
Months since diagnosis	11	(3, 19)
	*n*	(%)^a^
Number of participants	39	
Marital status		
Married/living together	23	(62)
Not partnered	14	(38)
Education		
Year 12 or below	24	(63)
TAFE/College/University	14	(37)
Private health insurance		
No	16	(41)
Yes	23	(59)
Country of birth		
Australia	31	(82)
Overseas	7	(18)
Health literacy (SILS)^b^		
Adequate	24	(75)
Limited	8	(25)
Prior personal history of cancer		
No	28	(72)
Yes	11	(28)
Comorbidities^c^		
None	7	(18)
One or more	32	(82)
Disease stage at diagnosis^d^		
I/II	23	(59)
III/IV	6	(15)
Unable to assess	10	(26)
Time since receiving last treatment		
Still receiving treatment	9	(26)
< 6 months ago	12	(34)
6 months to < 1 year ago	8	(23)
1+ years ago	6	(17)
Treatment received		
None	np	(< 10)
Surgery only	23	(62)
Surgery and adjuvant therapy	6	(16)
Adjuvant therapy only	np	(< 20)

*SILS*, Single Item Literacy Screener [15]; *np*, not publishable (cell count suppressed due to small numbers)

^a^Prevalence among non-missing observations (frequency of missing ranges 0 to 7)

^b^Adequate = never or rarely need help reading printed health material; limited = sometimes, often, or always need help reading printed health material

^c^Comorbidities include diabetes/high blood sugar, high blood pressure/hypertension, heart conditions, lung conditions, kidney disease, and depression/anxiety disorders

^d^Source: Queensland Cancer Register

### Satisfaction with information

The proportion of participants who reported being satisfied with the amount and helpfulness of information that they received increased from pre-diagnosis to during diagnosis (amount: 8/30, 27% to 23/36, 64%; helpfulness: 7/29, 24% to 22/35, 63%, respectively) and decreased slightly from diagnosis to during treatment (19/32, 59% and 18/32, 56%, respectively) (Table 2).

**Table 2:** Satisfaction with information during pre-diagnosis, diagnosis, and treatment

SCIP-B items^a^ and total scores	Pre-diagnosis		Diagnosis		Treatment	
	Mean	(SD)	Mean	(SD)	Mean	(SD)
Usefulness of information to you	3.0	(1.0)	3.6	(1.3)	3.7	(1.2)
Usefulness of information to your partner/family	3.1	(1.1)	3.4	(1.3)	3.3	(1.2)
Amount of written information	3.0	(1.1)	3.1	(1.3)	3.3	(1.3)
Amount of verbal information	3.0	(1.1)	3.5	(1.4)	3.8	(1.3)
Timing of which you found information	2.9	(1.1)	3.4	(1.4)	3.6	(1.2)
Amount of detail of the information	2.9	(1.1)	3.5	(1.4)	3.6	(1.3)
How understandable the information was to you	3.0	(1.1)	3.4	(1.4)	3.4	(1.4)
*Total satisfaction, mean (SD)*^*b*^	20.8	(7.1)	24.0^c^	(8.4)	24.8^c^	(8.2)
	***n***	(%)	***n***	(%)	***n***	(%)
Overall satisfaction with the amount of information^d^	8	(27)	23	(64)	19	(59)
Overall satisfaction that the information was helpful^d^	7	(24)	22	(63)	18	(56)

*SCIP-B*, Satisfaction with Cancer Information Profile [12]; *SD*, standard deviation

^a^Higher scores indicate higher satisfaction (range 1 = very dissatisfied to 5 = very satisfied); results among non-missing observations (range 2 to 11 missing observations)

^b^Based on the seven-item SCIP-B scale (range 7 to 35 where higher scores indicate higher satisfaction); results among non-missing observations (range 4 to 10 missing observations)

^c^Linear mixed model *p*-value < 0.05 compared to pre-diagnosis

^d^Overall satisfaction items are study-specific; prevalence of satisfied/very satisfied with information among non-missing observations (range 3 to 10 missing observations)

**Fig. 1:**
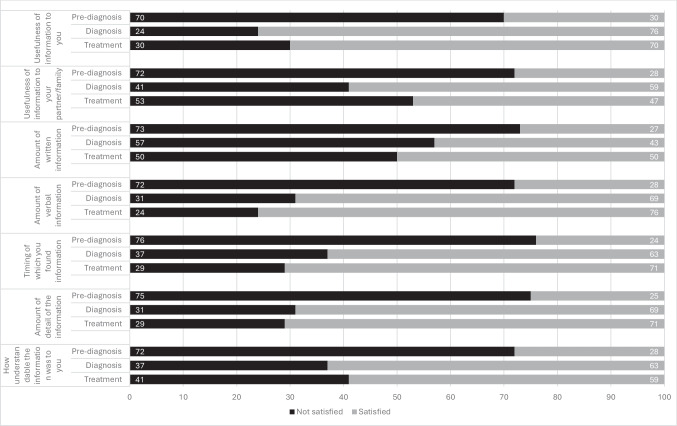
Prevalence^a^ of satisfaction with information during pre-diagnosis, diagnosis, and treatment. ^a^Prevalence among non-missing observations (range 2 to 11 missing observations); satisfied (4–5) and not satisfied (1–3) as measured on the Satisfaction with Cancer Information Profile (SCIP-B) [12]

There was an increase in the mean score for total satisfaction with information (SCIP-B) from pre-diagnosis (20.8, SD 7.1) to during diagnosis (24.0, SD 8.4) and treatment (24.8, SD 8.2) (Table 2). This increase was clinically (MID > 3) and statistically (*P* < 0.05) significant. As shown in Fig. 1, the proportion of participants who reported that they were not satisfied with the type and timing of individual information items was highest pre-diagnosis (range 21/30, 70% to 22/29, 76%). This proportion decreased during diagnosis (range 9/37, 24% to 20/35, 57%) and treatment (range 8/27, 24% to 17/32, 53%). The proportion of participants who were not satisfied was highest pre-diagnosis for timing of information (22/29, 76%) and amount of detail (21/28, 75%). During diagnosis and treatment, prevalence of not being satisfied was highest for the amount of written information (20/35, 57% and 16/32, 50%, respectively) and usefulness of information for their partner/family (14/34, 41% and 17/32, 53%, respectively) (Fig. 1).

### Unmet information needs

**Fig. 2:**
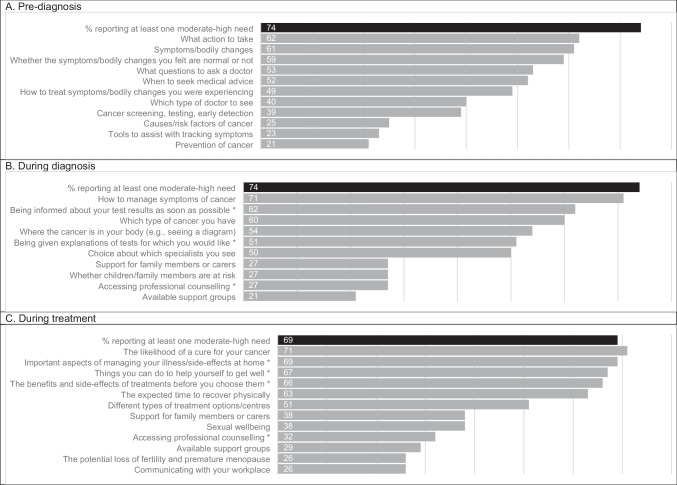
Prevalence^a^ of moderate-to-high unmet information need items during **A** pre-diagnosis, **B** diagnosis, and **C** treatment. ^a^Prevalence among non-missing observations (range 2 to 9 missing observations). *Items from the Supportive Care Needs Survey (SCNS-SF34) [13]

Approximately three-quarters of participants reported at least one moderate-to-high unmet information need pre-diagnosis (28/38, 74%), during diagnosis (29/39, 74%), and during treatment (27/39, 69%) (Fig. 2). The most prevalent moderate-to-high unmet needs pre-diagnosis were for information about “what action to take” (21/34, 62%), “symptoms/bodily changes” (20/33, 61%), and “whether the symptoms/bodily changes you felt are normal or not” (20/34, 59%). During diagnosis, the top moderate-to high unmet needs were for information on “how to manage symptoms of cancer” (24/34, 71%), “being informed about your test results as soon as possible” (23/37, 62%), and “which type of cancer you have” (21/35, 60%). During treatment, the most common moderate-to-high unmet needs were for information about “the likelihood of a cure for your cancer” (25/35, 71%), “important aspects of managing your illness/side effects at home” (25/36, 69%), and “things you can do to help yourself to get well” (24/36, 67%) (Fig. 2).

### Preferred sources of receiving information

The most commonly reported preferred sources of information across pre-diagnosis, diagnosis, and treatment included a “doctor or healthcare provider” (24/36, 67%; 34/36, 94%; and 32/34, 94%, respectively) and an “internet search” (20/36, 56%; 20/36, 56%; and 18/34, 53%, respectively) (Table 3). “Family and friends” were among the top three preferred sources pre-diagnosis and during diagnosis (12/36, 33% and 14/36, 39%, respectively), and “printed materials” was in the top three preferred sources during treatment (12/34, 35%) followed by “family and friends” (11/34, 32%) (Table 3).

**Table 3:** Women’s preferred sources of receiving information pre-diagnosis, during diagnosis, and during treatment (top three shown in bold)

Source of information^a^	Pre-diagnosis		Diagnosis		Treatment	
	*n*	(%)^b^	*n*	(%)^b^	*n*	(%)^b^
Doctor or healthcare provider	**24**	**(67)**	**34**	**(94)**	**32**	**(94)**
Internet search	**20**	**(56)**	**20**	**(56)**	**18**	**(53)**
Family or friends	**12**	**(33)**	**14**	**(39)**	11	(32)
Printed materials	5	(14)	10	(28)	**12**	**(35)**
Support group	4	(11)	2	(6)	0	(0)
Cancer organisation telephone information and support line	3	(8)	3	(8)	2	(6)
Government health agencies	3	(8)	1	(3)	2	(6)
Social media	3	(8)	2	(6)	1	(3)
Books	2	(6)	1	(3)	2	(6)
Complementary or alternative practitioner	1	(3)	3	(8)	2	(6)
Library	1	(3)	1	(3)	0	(0)
Magazines or newspapers	1	(3)	1	(3)	1	(3)

*HINTS*, Health Information National Trends Survey [15]

^a^As measured by the HINTS

^b^Prevalence among participants with at least one response selected (4 without a response at one or more touchpoints). Multiple responses were allowed. Measured using the HINTS

### Participant-informed recommendations

Table 4 outlines participant-informed recommendations to improve information provision for those with vaginal/vulvar cancer. Seventeen recommendations derived across pre-diagnosis (*n* = 2), diagnosis (*n* = 2), treatment (*n* = 4), post-treatment (*n* = 2), and across touchpoints (*n* = 7).

**Table 4:** Participant-informed recommendations to improve information provision for women diagnosed with vaginal and vulvar cancers

Categories and sub-categories	Examples from participants (participant identifier^a^)	Recommendations
Pre-diagnosis		
Need for information	“They didn’t tell me that it [lichen sclerosus] was precancer that’s just me looking through Google and trying to read as much as I can about it. The last time I went down for a checkup, it is spreading. So, I’m treating a disease that I have no idea where it is because the picture that I got them to take of my vagina, to me just looks like a vagina. So I’m treating something that I have no symptoms and I’m just slathering steroid cream all over it and hoping I’m getting it. I will have more questions to ask about where I can get the help and support from to talk to someone about the lichens”. (P122)	Offer women with high-risk conditions for developing vaginal or vulvar cancer clear information about their risks, treatment, cancer prevention, and sources of support
Need for information	“Just be aware, don’t ignore things that you know have changed in your body. I actually think there needs to be a programme, an ad on TV, where you say, ‘have you checked this, have you reminded your doctor you are due for your [checks]’”. (P195)	Health promotion initiatives should be designed and implemented to educate women on the signs and symptoms of vaginal and vulvar cancers
Diagnosis		
Need for information	“People with urinary tract infections, the number of times the doctor doesn’t even look at that part of our anatomy; they take a blood test and a urine test and then they give you a prescription”. (P111) “When ladies go in with an itch, they need to be biopsied for that and then checked for cancer”. (P133) “When women have pap smears that’s the opportunity for the doctor to go that one step further and explain to women even though it’s unusual this can happen [vulva melanoma]. Next time you have a pap smear go that one step further [a physical examination]. A two-minute inspection could save your life”. (P195) “The only time a GP would look at that part of our anatomy is for a smear test but then once you’ve had a hysterectomy, you don’t have to have smear tests”. (P111)	Provide information/education to women and healthcare professions about what is optimal care, including relevant investigations for when women present with gynaecological concerns, for cervical screening, and for women who have had a hysterectomy
Sources of information	“[My sister] recommended to look up Australian websites if I do need to. So when I did get the diagnosis, I started looking up the Australian websites like the Cancer Council”. (P107)	Direct people to reliable, Australian websites
Treatment		
Satisfaction with information	“That would be a good thing, to have a pamphlet to give a few little things like that to the patient beforehand, just so that they know what to expect. Medically, I imagine that those details would be quite minor (e.g., pads, toilet, wee going everywhere)”. (P128)	Information about potential treatment side effects and how to manage them should be communicated ahead of time so that women know what to expect
Need for information	“I think they could probably do a lot more in the booklet after surgery. The booklet that we got was just like a general after-surgery booklet. Not specific to having vulvar cancer surgery. I think that would have answered some of the questions that I had at that time”. (P122)	Provide information relevant to the specific cancer surgery
Need for information	“I don’t know if a lot of patients would know. For regional people if your surgery, your treatment, has to be done in Brisbane or in regional Queensland you sign up for this patient travel expenses and accommodation expenses, so they assist you with paying for fuel, accommodation, for a support person as well to be with you. It helps with financial distress”. (P127) “If patients have some sort of income insurance, to put that in motion as early as they can”. (P127)	Provide people with information about available financial support
Sources of information	“When you go and have your treatment, your radiation treatment, at the end of every session instead of just saying, ‘Oh, I’ll see you tomorrow’, having a visit every now and then. I think I had one with the doctor before I started”. (P167)	Offer regular check-ins with the treating doctor as part of ongoing care to enable information exchange between patient and the treating doctor
Post-treatment		
Satisfaction with information	“They did say [when I was discharged] that I would have to come in for a checkup at two months, but they didn’t tell me every two months for the next two years that I’m gonna have to go for a check-up down in Brisbane. They didn’t tell me they were gonna do a biopsy. That’s when the doctor then explained I need to come every two months for two years because in those first two years is when the cancer can come back. That’s just a bit of common courtesy. Just letting you know, apart from everything else that’s going on, this is just gonna be part of your life for the next two years. Sometimes when life happens you go, oh, I’m just not gonna go to that appointment, if I don’t understand why”. (P122)	Information about the follow-up frequency, work-up, and explanation for this should be communicated ahead of time
Need for information	“No one can tell me. They can’t tell you how long you’ve got. They can give you a ballpark figure but we just don’t know. I don’t see it as, as morbid, but I do see it as, as realistic”. (P168)	Doctors should provide clear and accurate information on the likelihood of a cure, recurrence, and life expectancy
Across touchpoints		
Need for information	“It is pretty embarrassing to talk about. None of my friends have ever heard of vulvar cancer. I think it needs to be out there more. But it is quite embarrassing to talk about, especially to males [friends, family, partner]”. (P107)	Community-based awareness initiatives should aim to improve awareness of vaginal and vulvar cancers and reduce stigma so that women feel less embarrassed and better supported by their networks
Need for information	“The Cancer Council do have their list of all of the different cancers but the information is very basic. If you’ve got something that’s a little bit different and a little bit rare there’s not much out there at all”. (P104) “I think there is some really great information that I was given but it was about cervical cancer and you’re just told to cross that out and put vagina. It doesn’t mean the same when you have to just substitute one word for the other when they’re different parts of the body”. (P168)	Specific information relevant to rare gynaecological cancers is needed
Need for information	“Regional people need more information – they don’t get as much information out there. Often, a lot of information doesn’t reach them”. (P127)	Improve the availability and accessibility of information for people living in rural and remote areas
Need for information	“I went into the gyno to get the biopsy done and one of the gynos there said, ‘Oh, you’re the second person who’s had … cancer’ and I said ‘Who? Where is she? Point her out. Does she wanna talk?’ She had already gone through it – it would’ve been nice”. (P104) “It would’ve been nice to have on that [hospital] form where I could have gone and got support from people to talk about the cancer that I’d had and the surgery that I had”. (P122)	Offer information on how to access peer support and connect with others with the same cancer
Sources of information	“You need more detailed information from doctors and not from the internet. Because the internet, a lot of it is so deceiving”. (P133)	Clear and detailed information should be provided by doctors throughout the care pathway
Sources of information	“The Cancer Council has quite a lot of information on it. They show you pictures of vulvectomies and that sort of stuff and I had a partial vulvectomy”. (P101)	Direct people to the Cancer Council website
Sources of information	“The last thing you want is to bring home loads of brochures. You don’t want that to be seen anywhere and so a QR code where they can access different things”. (P127)	Use QR codes to support access to information—instead of, or alongside, printed brochures

^a^This study recruited participants with vaginal and vulvar cancer simultaneously with those with a different cancer type. Original identifiers have been retained, and therefore, participant identifier numbers do not total to 39

Women indicated that in the pre-diagnosis period, there was a need for better information about high-risk conditions for developing vaginal or vulvar cancer and the signs and symptoms of these cancers. During the diagnosis period, they wanted to know more about optimal care and relevant investigations and reliable internet sources of information. During treatment, women indicated that information needed to be provided earlier, so they knew what to expect in advance (e.g. treatment side effects). Women specifically identified the need for information about their cancer surgery and financial support services, including funding for rural/remote residents to travel for treatment. Timing of information was also important post-treatment (e.g. about frequency of follow-up appointments), and the need for specific prognosis information was often discussed.

## Discussion

In this study of Queensland women with vaginal and vulvar cancer, many (> 70%) participants were not satisfied with information received pre-diagnosis, and moderate-to-high unmet information needs are experienced by the majority of women at each touchpoint (~ 75%). Interviews captured a range of participant-informed recommendations which could guide efforts to improve information provision for women with vaginal and vulvar cancers.

Our study is the first to explore cancer information experiences pre-diagnosis. While satisfaction with information was low pre-diagnosis, this improved during diagnosis and treatment with a similar prevalence for being satisfied (56%–64%) as previously reported among endometrial (60%) and ovarian (65%) cancer survivors [25, 26]. A survey conducted among people with rare solid tumours showed slightly higher prevalence of satisfaction with information (> 70%) concerning their disease treatment, effects of cancer medication, side effects, and support [27]. That study also showed that satisfaction with information varied by patients of different rare cancer types [27].

A comparison with the literature shows that many of the unmet needs identified in the current study are commonly reported. Similar to our findings, previous studies found that common unmet information needs among patients with pancreatic, ampullary, gynaecological, and advanced cancers include information on what to expect, how to stay well, test results, remission/prognosis, and managing side effects [25, 28–30]. A systematic review of unmet supportive care needs of patients with rare cancer found the most frequently reported unmet needs were in the “healthcare system and information” domain and these were experienced more often compared to patients with common cancer [5]. Furthermore, of the eight studies among people with rare gynaecological cancers, none reported on unmet needs in the diagnostic phase [5].

The preferred sources of information reported by participants in the current study (healthcare professionals, the internet, family/friends, and printed materials) also appear consistent with previous research among gynaecological cancer patients and adult patients more broadly [31, 32].

There are several plausible reasons why women report low satisfaction and prevalent unmet information needs in relation to their cancer diagnosis and treatment. Healthcare professionals, reported by participants as the top preferred source of information, may not be aware of patients’ specific information needs, and this could create a mismatch between patient needs and what healthcare professionals perceive as important [33, 34]. Healthcare professionals may not feel competent to communicate sensitive information to gynaecological cancer patients on, for example, sexual health [33]. Alternatively, healthcare professionals may provide information, but the patient may not recall receiving it because of anxiety, treatment side effects, or information overload [35, 36].

Our results extend the previous limited research on information experiences specifically for women diagnosed with vaginal and vulvar cancers. While women articulated a desire to be better informed throughout the cancer journey, this study provides insight into a historically under-researched phase of the care journey: pre-diagnosis [5]. Additionally, we provide participant-informed recommendations, which highlight a key message: simply providing more information may not be effective on its own; rather, focusing on the quality of information and communication, appropriateness of timing, and the trustworthiness of the “messenger” is likely to be crucial to improving information experiences. This study provides a guide on the types of information patients with vaginal or vulvar cancer want and when they would prefer to receive this information. While participants did not identify *who* is responsible for enacting each of the recommendations, this is likely to be a collective effort.

Based on our results, practical implications for healthcare professionals to improve information provision might include providing printed information for patients and family to take away and directing them to trusted online information sources. Participant-informed recommendations could also be used to tailor and test interventions to improve information experiences. A systematic review found that interventions to improve cancer patients’ information needs and satisfaction via face-to-face communication and written material delivered by a healthcare professional are promising; however, similar interventions targeting rare cancer survivors are limited [37]. Of 34 studies included in the review, one study included participants with ovarian and vaginal cancer [37].

Our recommendations may also contribute to improving information provision among other rare cancer patients. It is likely that many of the information challenges women with vaginal/vulvar cancer experience across the cancer care continuum are shared with other rare cancers, such as the delay in obtaining the correct diagnosis, lack of disease-specific and treatment-specific information, lack of clear cancer care pathways, limited specialists, and specialist centres. However, recommendations are likely to need tailoring, e.g. by age, and side effects are likely to differ depending on the treatment types and anatomical location.

### Limitations

While our study explored patient experiences of the information pathway from pre-diagnosis to treatment, we did not ask about post-treatment information experiences in the survey. This omits the survivorship phase of the cancer care continuum. However, participants were not limited by this during interviews, and we know much more about survivorship than earlier phases of the cancer continuum [38]. Results should be interpreted with caution given the low response proportion and small sample size. Measured characteristics of participants and non-participants were similar, but we may not have captured the full spectrum of information experiences across all women with vaginal and vulval cancer. We also excluded older women and those living in a nursing home who may have different information experiences. Additionally, we asked participants to recall pre-diagnosis, diagnosis, and treatment periods which for some were many months ago and thus may differ by touchpoint for individuals. Finally, while we recruited via a population-based registry and across remoteness and socio-economic areas in Queensland, the small sample size limits the generalisability of our findings.

## Conclusions

Our cross-sectional, mixed methods study indicated that while information experiences among vaginal and vulvar cancer patients in Queensland are varied, there is room for improvement. The results of this study should be interpreted as a broad perspective of experiences that could be faced by vaginal/vulvar cancer survivors along the cancer care continuum. This study has identified areas in which some women with vaginal/vulvar cancer are dissatisfied with information received and areas where a perceived need for information goes unmet. The findings include participant-informed recommendations which should be explored to address these gaps. Further in-depth analysis of the qualitative data will help to elaborate on these findings and guide future research, using co-design principles with consumers with lived experience and clinicians, to identify when and how to implement recommendations to improve information provision along the care pathway.

## Supplementary Information

Below is the link to the electronic supplementary material.

ESM 1 (DOCX 89.7 KB)

## Data Availability

The data for this study will not be shared, as we do not have permission from the participants or ethics approval to do so.
