# Supplementary material for: Vaginal and vulvar cancer patient experiences of the information pathway from pre-diagnosis to treatment
Source: Support Care Cancer. 2026 Jul 21;34(8):787. doi: 10.1007/s00520-026-11022-0 (PMC13388510; doi:10.1007/s00520-026-11022-0)
Supplement: Supplementary file 1 — (DOCX 89.7 KB) [file 520_2026_11022_MOESM1_ESM.docx]

Supplementary Material 1. Interview guide and journey map

General questions for the interview:

- The questionnaire you completed asked about your information needs during your cancer journey. Tell me about your own cancer journey, from early on until now.
- Describe your personal experiences as a series of steps.
- Touchpoints along the cancer journey
  - What were you doing/actions taken during this time?
  - What were you thinking during this time?
  - Who did you speak to during this time?
  - What were you feeling during this time?
- Tell me about your information needs and experiences.
- Tell me about your communication needs and experiences.
- Were there aspects of information/communication that were particularly helpful?
- Were there aspects about the information/communication that you would change if you could?

Journey map:

**
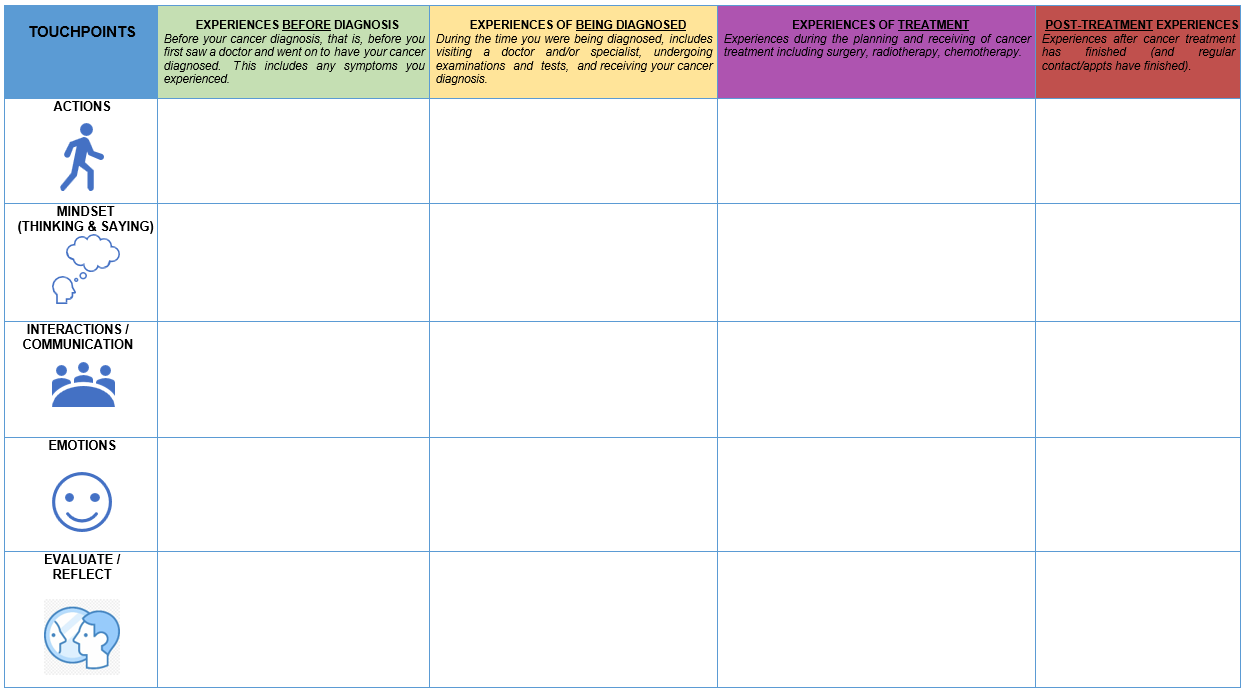
**

Supplementary Table 1. Additional table comparing total SCIP-B scores (mean, SD) by analytical approaches

| Touchpoint | Raw summary of all available data | Raw summary of those with complete data only (n=26) | Mixed model (unstructured) | Mixed model (unstructured) with age |
| --- | --- | --- | --- | --- |
| Pre-diagnosis | 20.8 (7.1) | 21.3 (7.0) | 20.9 (18.5, 23.3) | 20.9 (18.5, 23.2) |
| Diagnosis | 24.0 (8.4) | 23.5 (8.6) | 24.2 (21.5, 26.6) | 24.1 (21.6, 26.6) |
| Treatment | 24.8 (8.2) | 24.2 (8.3) | 24.6 (21.6, 27.6) | 24.7 (21.6, 27.7) |
| SCIP-B: Satisfaction with Cancer Information Profile.^12^ | | | | |

Supplementary Table 2. Comparison of characteristics between study participants and non-participants

| Characteristics^a^ | Participants | | Non-participants | |
| --- | --- | --- | --- | --- |
|  | n | (%) | n | (%) |
| Number of women | 39 | | 73 | |
| **Primary site** |  |  |  |  |
| Vaginal | 6 | (15) | 15 | (21) |
| Vulvar | 33 | (85) | 58 | (79) |
| **Age group at diagnosis** |  |  |  |  |
| <50 years | 5 | (13) | 11 | (15) |
| 50-69 years | 19 | (49) | 35 | (48) |
| >70 years | 15 | (38) | 27 | (37) |
| **Remoteness^b^ of residence at diagnosis** |  |  |  |  |
| Major city | 25 | (64) | 50 | (69) |
| Inner regional | 9 | (23) | 12 | (16) |
| Outer regional/remote/very remote | 5 | (13) | 11 | (15) |
| **Socio-economic status^c^** |  |  |  |  |
| Disadvantaged | np | (<20) | 15 | (21) |
| Middle | 28 | (72) | 48 | (66) |
| Affluent | np | (<15) | 10 | (14) |
| ^a^ Source: Queensland Cancer Register.  ^b^ Remoteness: The relative remoteness of residence at time of diagnosis is derived from the Australian Standard Geographical Classification (ASGC).^19^  ^c^ Socio-economic status: Based on the Socio-Economic Indexes for Areas (SEIFA).^20^  np. not publishable (cell count supressed due to small numbers). | | | | |
